# An ultra-low crosstalk and broadband two-mode (de)multiplexer based on adiabatic couplers

**DOI:** 10.1038/srep38494

**Published:** 2016-12-06

**Authors:** Chunlei Sun, Yu Yu, Mengyuan Ye, Guanyu Chen, Xinliang Zhang

**Affiliations:** 1 Wuhan National Laboratory for Optoelectronics, School of Optical and Electronic Information, Huazhong University of Science and Technology, Wuhan, 430074, China

## Abstract

A novel adiabatic couplers (ACs) based broadband and fabrication-tolerant two-mode multiplexer (MUX) is designed using silicon-on-insulator (SOI) platform. Being different from the previously reported ACs-based scheme, the converted and multiplexed signals are on conventional modes, rather than supermodes. The experimental results are in good agreement with the simulations. Over a wavelength range of 75 nm measured, the crosstalk is lower than −20 dB, and the insertion loss is ~1 dB. The eye diagram and bit error rate measurements validate the good performance of the proposed mode MUX. The investigation on fabrication tolerance indicates reasonable performance degradation for a large gap deviation from −30 to 30 nm and etching depth deviation from −50 to 50 nm.

To satisfy the exponentially increasing data demand, photonic interconnections based on silicon-on-insulator (SOI) technology have been proposed as a promising solution and attracted significant interests in the past few years. On the other hand, various multiplexing techniques have been utilized to increase the capacity of optics communication link, such as wavelength-division multiplexing (WDM)^1^ and polarization-division multiplexing (PDM)^2^. More recently, optical communication using mode-division multiplexing (MDM) has also attracted lots of attentions, since it provides an effective method to further increase the transmission capacity. A high-performance mode multiplexer/demultiplexer (MUX/DeMUX) with low crosstalk, broad bandwidth, low insertion loss and large fabrication tolerance is the key component for realizing MDM link. Many mode MUX schemes had been proposed using SOI platforms, including asymmetric directional couplers (ADCs)^3^,^4^, multimode interference (MMI)^5^,^6^, adiabatic couplers (ACs)^7^,^8^,^9^ and asymmetric Y-junction^10^,^11^. The ADC-based devices require precise phase matching and are therefore inherently sensitive to fabrication errors. The design process of MMI-based devices is usually complicated and a phase shifter sensitive to fabrication errors needs to be cascaded. For asymmetric Y-junction, very precise fabrication is usually required to obtain the desired ultra-small gaps. Conversely, the ACs-based devices benefit from broad bandwidth and large fabrication tolerance due to its operation principle of mode evolution.

In this study, we propose and demonstrate a novel two-mode ACs-based MUX/DeMUX. Being different from the previously reported ACs-based scheme^7^, the output waveguide is a conventional multimode bus waveguide rather than a slot waveguide. In ref. 7, the slot waveguide supporting supermodes is not compatible with conventional multimode waveguide, disabling the integration compatibility with other multimode devices based on conventional mode, such as higher order-mode pass filter^12^. Furthermore, the slot waveguide is difficult to achieve low propagation loss^13^,^14^, and must be carefully etched to form a lower and uniform gap along the transmission path. In our device, a specifically designed mode converter consisting of a power splitter and a Y-junction can convert the even and odd supermodes into the corresponding conventional modes of a multimode bus waveguide. The experimental results are in good agreement with the simulations obtained. Experiment results show that the crosstalk is lower than −20 dB, and the insertion loss is ~1 dB over a wavelength range of 75 nm. The non-return-to-zero on-off-keying (NRZ-OOK) signal at 40 Gb/s is used to test the proposed scheme, indicating a power penalty less than 1 dB for the whole MDM link. The fabrication tolerance is also characterized, and results show a good fabrication tolerance on gap and etching depth.

## Results

### Principle and simulation

**Figure 1:**
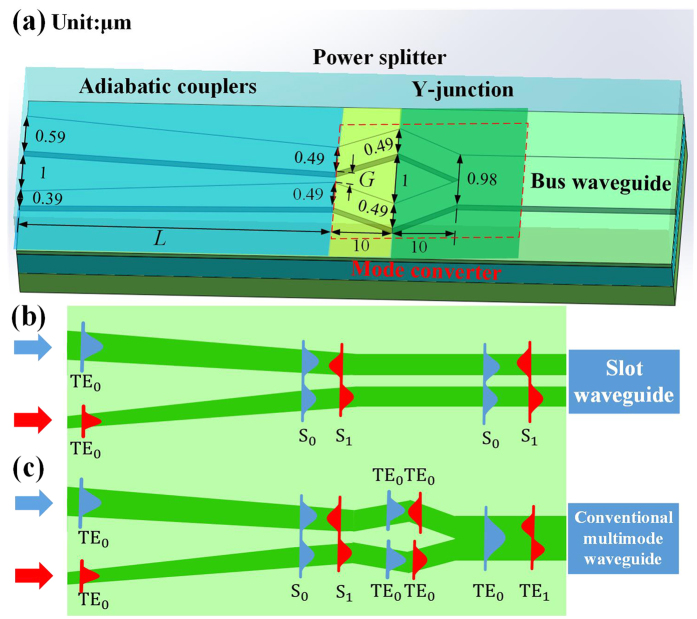
Schematic drawing and operation principle. (**a**) Schematic drawing of the proposed two-mode (De)MUX consisting of ACs and a power splitter and a symmetric Y-junction. Operation principle of (**b**) the previously reported AC-based and (**c**) our scheme.

Figure 1(a) shows the proposed mode MUX, which consists of ACs, a power splitter and a symmetric Y-junction. The operation principle relies on the mode evolution in the two waveguides forming the ACs. The two separate waveguides are firstly designed with different widths and a large gap, then they are tapered adiabatically to the same width and the gap is decreased gradually, forming a coupled-waveguide system with two system guided modes. Figure 1(b) and (c) show the operation principle of the previously reported and our ACs-based schemes, respectively. The ACs in the two schemes have the same geometry designs. The fundamental transverse electric (TE_0_) mode in the upper (lower) waveguide adiabatically evolves into a supermode S_0_ (S_1_), and then propagates in the slot waveguide in the previously reported scheme. However, in our scheme, the S_0_ (S_1_) mode is further processed by a power splitter, through evenly splitting power into two single-mode waveguides. The two split signals are both at fundamental mode but with zero or π phase difference for S_0_ or S_1_ input, respectively^15^. Generally speaking, the first order TE (TE_1_) mode can be effectively regarded as a combination of two antiphase TE_0_ modes^16^. Therefore, the two TE_0_ modes with π phase difference are combined and converted into a TE_1_ mode after propagating through a subsequent Y-junction. By contrast, the two TE_0_ modes with the same phase are merged into a TE_0_ mode, as shown in Fig. 1(c). Thus, the power splitter and Y-junction constitute a mode converter, converting the S_0_ and S_1_ modes into TE_0_ and TE_1_ modes, respectively. Thanks to the proposed mode converter, the mode MUX can be seamlessly connected with conventional multimode waveguide.

The proposed scheme is designed based on 220 nm SOI wafer, and the waveguide is rib one with 90 nm slab height. The entire device is covered by SiO_2_ cladding. For the ACs, the two waveguides are linearly tapered from 0.59 and 0.39 μm to 0.49 μm, respectively. The gap between the two waveguides is linearly tapered from 1 μm to G. Note that the two waveguides with different widths are firstly designed with a large gap to ensure the decoupling, avoiding unwanted interference. The width and length of the power splitter are 0.49 and 10 μm, respectively. The widths of the branch and stem parts of the Y-junction are 0.49 and 0.98 μm, while the branch length is 10 μm. The gap between the branches of Y-junction is set as 1 μm. To be noted, the angle between the branches of the Y-junction is 5.1 degrees, which is much larger than that of asymmetric Y-junction (~1 degree)^10^, alleviating the fabrication difficulty.

**Figure 2:**
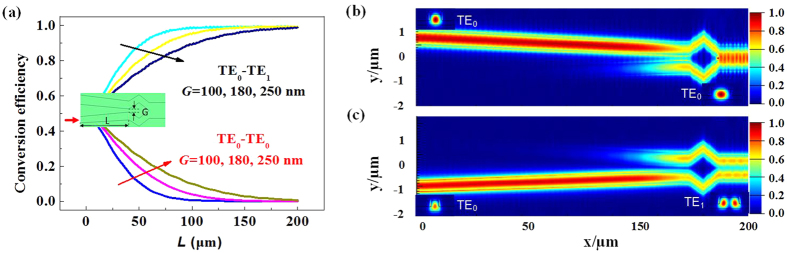
Conversion efficiency and field distributions. (**a**) Calculated conversion efficiency for the TE_0_ mode to ones in stem of Y-junction at 1550 nm as a function of the length of ACs (*L*) when the TE_0_ mode is launched from the lower waveguide (Here the gap *G* = 100, 180, 250 nm). Simulated field distributions when the TE_0_ modes are launched from the (**b**) upper and (**c**) lower waveguides at 1550 nm. Here the gap *G* = 180 nm, the ACs length *L* = 180 μm. The inserts show the mode distribution of each input and output ports.

Figure 2(a) shows the calculated conversion efficiency with different ACs length *L*, for the TE_0_ mode launched from the lower input waveguide at 1550 nm. Here we define the conversion efficiency as the ratio of the power of the TE_0_/TE_1_ mode in the multimode waveguide to the power of the TE_0_ mode injected at the upper/lower port of the ACs. Three different gaps (*G* = 100, 180 and 250 nm) are compared. Results reveal that the efficiency for TE_0_ to TE_1_ mode conversion can be very high (>95%) provided the ACs length *L* is designed to be larger than 150 μm. On the other hand, the efficiency increases with the reduction of gap *G* for a fixed length, and the efficiency difference is very small when the length is larger than 150 μm. Meanwhile, the crosstalk (TE_0_ to TE_0_) is extremely low. Same results can be obtained for the other case, i.e. inputting from the upper waveguide. In consideration of fabrication and footprint of the MDM link, *G* = 180 nm and *L* = 180 μm are utilized in our design. Figure 2(b) and (c) show the simulated field distributions of the proposed two-mode (De)MUX at 1550 nm, and the insets show the mode profiles at both ends. It can be seen that a TE_0_ mode will be obtained at the output when TE_0_ mode is launched from the wide (upper) input. The TE_0_ mode will be converted into a TE_1_ mode if injected from the narrow (lower) input. Thus, if both TE_0_ signals are injected from both input ports (upper and lower), the mode multiplexed signals (TE_0_ + TE_1_) can be obtained. Therefore, the multiplexing function is achieved at the stem of the Y-junction.

**Figure 3:**
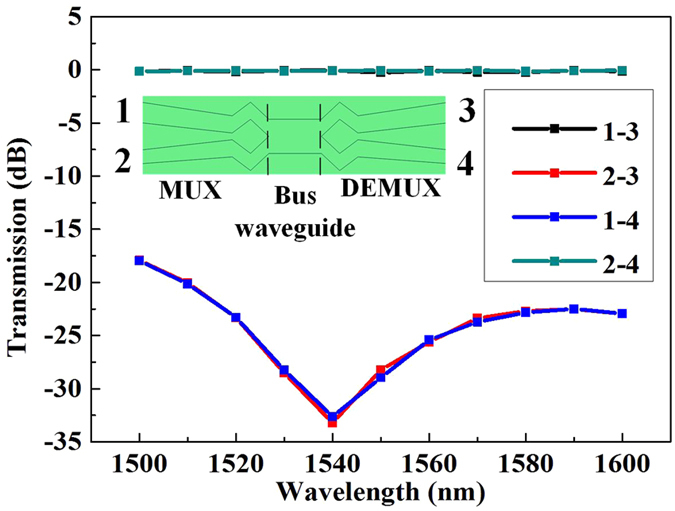
Calculated transmission spectra of the MDM link. Inset: the MDM link covering the mode MUX, the bus waveguide and the mode DeMUX. The length of the bus waveguide is 30 μm.

**Figure 4:**
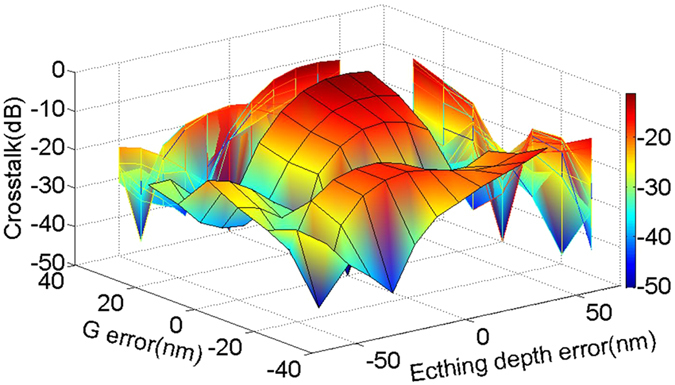
Fabrication tolerance analysis. 2D sweeping of crosstalk versus gap G and etching depth errors.

The wavelength dependence of the proposed MDM link is calculated to investigate the bandwidth, with results shown in Fig. 3. The schematic drawing of the full MDM link (including a MUX and a DeMUX) is presented as the inset, with two input ports 1 & 2 and two output ports 3 & 4. The symmetrical MUX and DeMUX, as well as a straight bus waveguide with 30 μm length, are designed. The crosstalk lower than −18 dB and insertion loss less than 0.3 dB for both modes over a broad range from 1500 to 1600 nm are realized. The valley of the crosstalk curves around 1540 nm can be attributed to self-imaging within the multimode bus waveguide^17^. This is the mode interference due to imperfect excitation of the mode MUX, producing the same optical profile on both interfaces of the bus waveguide. Note that the position of the crosstalk valley is dependent on the length of the multimode bus waveguide. In Fig. 4, the crosstalk versus the gap *G* and the etch depth errors are calculated at 1550 nm, respectively. It can be seen that the crosstalk is mostly lower than −20 dB. The crosstalk is ~−12 dB when the gap G error is 30 nm and etching depth error is 50 nm, which is within the reasonable degradation range. On the other hand, the fabrication error of wafer thickness is as low as 10 nm, and the crosstalk can keep ~−25 dB within 10 nm wafer thickness deviation. Obviously, a large fabrication tolerance can be observed, indicating a good reliability of the proposed scheme.

### Experimental results

**Figure 5:**
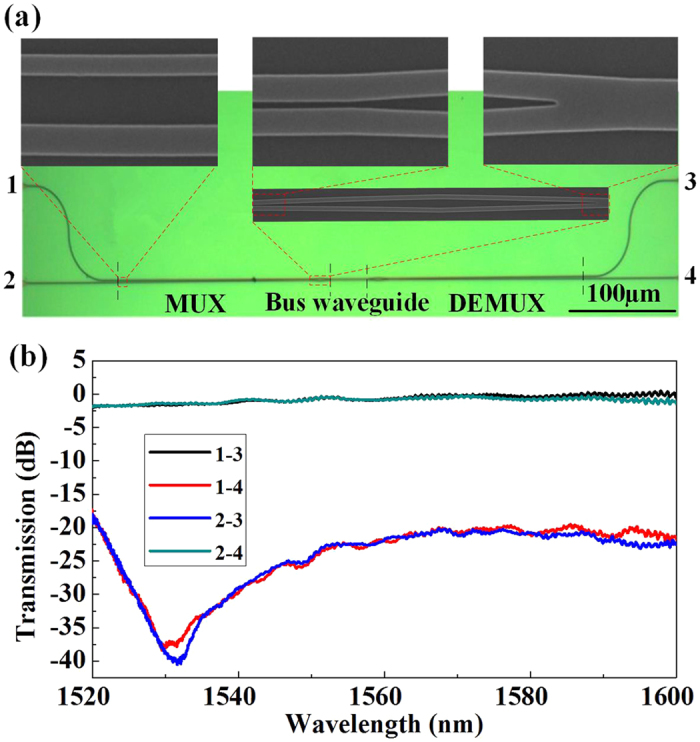
Pictures and performances of the fabricated MDM link. (**a**) Microscope and SEM top views. (**b**) Measured normalized transmission spectra.

Figure 5(a) shows the microscope and scanning electron microscope (SEM) top views of the fabricated MDM link, including the mode MUX, DeMUX and the multimode bus waveguide. The zoom-in pictures of the ACs, the power splitter and the Y-junction are also presented as insets of Fig. 5(a). In order to verify the performance of the fabricated MDM link, the transmission spectra are measured using a broadband source. A TE grating coupler (GC) is adopted for fiber-chip coupling. By subtracting the loss caused by the GCs, we get the actual response of the device. The measured crosstalk lower than −20 dB and insertion loss ~1 dB can be obtained over a range of 75 nm from 1525 to 1600 nm, as presented in Fig. 5(b). It is worth mentioning that fiber chip coupling loss is high and the output power from the broadband light source is very low around 1530 and 1600 nm. The actual bandwidth for crosstalk lower than −20 dB is larger than 75 nm. The measured results accord well with the simulation.

**Figure 6:**
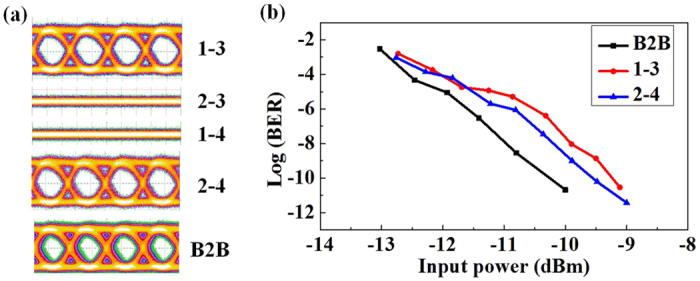
Signal test of the proposed scheme. (**a**) Measured eye diagrams at 1550 nm. (**b**) BER measurements for back-to-back (B2B) test case, port 1 to 3 and port 2 to 4 at 1550 nm.

The non-return-to-zero on-off-keying (NRZ-OOK) signal at 40 Gb/s is used to test the proposed scheme. The eye diagrams of the signals from both port 3 and 4 are measured in Fig. 6(a), showing that clear and open eyes can be obtained at one output port while signal from the other output port can be barely detected. These results indicate a good performance of the proposed device. Finally, the bit error ratio (BER) measurements are performed for the cases of port 1 to 3 and port 2 to 4, respectively. The results are plotted in Fig. 6(b). The back-to-back case is also measured as a reference, and the measured power penalties are both less than 1 dB. Note that the device is actually characterized by one channel at a time due to the limited experimental facility. Fortunately, the fabricated device exhibits a very low crosstalk, as the measured results shown in Fig. 5(b). Thus, the power penalty will not be increased obviously, if we were to transmit signal on both modes at the same time.

## Discussion

In summary, we have demonstrated a broadband and fabrication-tolerant two-mode (De)MUX scheme consisting of ACs and a specially designed mode converter. The proposed mode converter bridges the waveguides supporting supermode and conventional mode. The MDM link with proposed MUX/DeMUX is measured and demonstrated with crosstalk lower than −20 dB and insertion loss ~1 dB over a wavelength range of 75 nm. The NRZ-OOK signal at 40 Gb/s is used to test the proposed MDM link, indicating a good performance with power penalty less than 1 dB. The fabrication tolerance is also investigated, showing reasonable performance degradation for a large gap deviation from −30 to 30 nm and etching depth deviation from −50 to 50 nm. Actually, the proposed scheme is not easy to scale up for more modes due to its special geometry. However, even two-mode multiplexer also is still meaningful to increase the communication capacity. Extensive investigations on two-mode MUXs had been given to intra-chip interconnect^5^,^6^,^10^. Moreover, by combination our proposed MUX with other mode MUX such as asymmetry-directional-couplers-based one, the more modes multiplexing can be expected. On the other hand, coupling between few-mode fiber (FMF) and chip also have been widely investigated^18^,^19^, enabling compatibility of the proposed MUX with the SDM in fiber.

## Methods

### Simulation method

The properties (electric field distributions and transmission spectra) of the proposed two-mode (de)multiplexer based on adiabatic couplers are calculated by a eigenmode expansion (EME) solver. The scattering bound condition is considered and the simulation domain is surrounded by rectangular perfectly matched layer (PML).

### Device fabrication and experimental method

**Figure 7:**
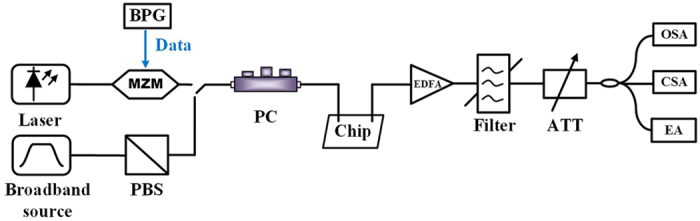
Experimental setup. BPG: bit pattern generator; OSA: optical spectrum analyzer; CSA: communications signal analyzer; EA: error analyzer.

The device is fabricated utilizing 248 nm deep ultraviolet photolithography and inductively coupled plasma (ICP) etching using SOI wafer with top silicon layer of 220 nm and silicon dioxide (SiO_2_) substrate of 2 μm. The etched structures have a SiO_2_ cladding layer by utilizing plasma-enhanced chemical vapor deposition. In order to obtain the actual response of the device, a silicon waveguide directly connected by a pair of GCs are also fabricated as references. The modulated signal at 40 Gb/s is used to further test the proposed device, and the experimental setup is shown in Fig. 7. A CW laser at 1550 nm is launched into the Mach-Zehnder modulator (MZM) to obtain the NRZ-OOK signal (2^31^-1 pseudo-random binary sequences). Being assisted by the polarization controller (PC), the maximum coupling efficiency of the grating coupler can be achieved. The erbium-doped fiber amplifier (EDFA) and attenuator (ATT) are utilized to optimize the output power.

## Additional Information

**How to cite this article**: Sun, C. *et al*. An ultra-low crosstalk and broadband two-mode (de)multiplexer based on adiabatic couplers. *Sci. Rep.* **6**, 38494; doi: 10.1038/srep38494 (2016).

**Publisher's note:** Springer Nature remains neutral with regard to jurisdictional claims in published maps and institutional affiliations.
